# An Outlook on Global Regulatory Landscape for Genome-Edited Crops

**DOI:** 10.3390/ijms222111753

**Published:** 2021-10-29

**Authors:** Aftab Ahmad, Nayla Munawar, Zulqurnain Khan, Alaa T. Qusmani, Sultan Habibullah Khan, Amer Jamil, Sidra Ashraf, Muhammad Zubair Ghouri, Sabin Aslam, Muhammad Salman Mubarik, Ahmad Munir, Qaiser Sultan, Kamel A. Abd-Elsalam, Sameer H. Qari

**Affiliations:** 1 Center for Advanced Studies in Agriculture and Food Security (CASAFS), University of Agriculture, Faisalabad 38000, Pakistan; aftab.ahmad@uaf.edu.pk (A.A.); sultan@uaf.edu.pk (S.H.K.); zubair.ghouri@pern.onmicrosoft.com (M.Z.G.); sabinaslam@gmail.com (S.A.); msmubarik@gmail.com (M.S.M.); qaisar.sultan1992@gmail.com (Q.S.); 2 Department of Biochemistry, University of Agriculture, Faisalabad 38000, Pakistan; amerjamil@yahoo.com (A.J.); sidra.best09@gmail.com (S.A.); amunir14046@gmail.com (A.M.); 3 Department of Chemistry, United Arab Emirates University, Al-Ain 15551, United Arab Emirates; nmunawar@uaeu.ac.ae; 4 Institute of Plant Breeding and Biotechnology, MNS University of Agriculture Multan, Multan 60000, Pakistan; zulqurnain.khan@mnsuam.edu.pk; 5 Biology Department, Al-Jumum University College, Umm Al-Qura University, Makkah 24243, Saudi Arabia; atqumsani@uqu.edu.sa; 6 Center for Agricultural Biochemistry and Biotechnology (CABB), University of Agriculture, Faisalabad 38000, Pakistan; 7 Plant Pathology Research Institute, Agricultural Research Center (ARC), Giza 12619, Egypt; kamelabdelsalam@gmail.com; 8 Molecular Biology Central Laboratory (GMCL), Department of Biology/Genetics, Aljumum University College, Umm Al-Qura University, Makkah 24243, Saudi Arabia

**Keywords:** genome editing 2, CRISPR/Cas 3, global regulations 4, CRISPR crops

## Abstract

The revolutionary technology of CRISPR/Cas systems and their extraordinary potential to address fundamental questions in every field of biological sciences has led to their developers being awarded the 2020 Nobel Prize for Chemistry. In agriculture, CRISPR/Cas systems have accelerated the development of new crop varieties with improved traits—without the need for transgenes. However, the future of this technology depends on a clear and truly global regulatory framework being developed for these crops. Some CRISPR-edited crops are already on the market, and yet countries and regions are still divided over their legal status. CRISPR editing does not require transgenes, making CRISPR crops more socially acceptable than genetically modified crops, but there is vigorous debate over how to regulate these crops and what precautionary measures are required before they appear on the market. This article reviews intended outcomes and risks arising from the site-directed nuclease CRISPR systems used to improve agricultural crop plant genomes. It examines how various CRISPR system components, and potential concerns associated with CRISPR/Cas, may trigger regulatory oversight of CRISPR-edited crops. The article highlights differences and similarities between GMOs and CRISPR-edited crops, and discusses social and ethical concerns. It outlines the regulatory framework for GMO crops, which many countries also apply to CRISPR-edited crops, and the global regulatory landscape for CRISPR-edited crops. The article concludes with future prospects for CRISPR-edited crops and their products.

## 1. Introduction

Agriculture is facing global challenges arising from the planet’s rapidly growing human population, expected to reach nine billion by 2050 [1]. Global climate change and rising biotic and abiotic stresses are also placing increased pressure on agricultural production. Food prices, especially in less-developed countries, are continually increasing and will likely keep doing so. In order to feed a larger population and address growing demands for better nutrition, agriculture must produce more using the same finite land areas and resources. Fresh water resources are declining worldwide. If we are to save precious water resources and reduce carbon emissions, agricultural practices must change. Climate-resilient crop varieties with enhanced yield and improved response to biotic stress are also needed. Swift action is required to address global food security challenges [2,3].

New crop varieties have traditionally been produced by conventional plant breeding methods. For example, cross-pollination can produce offspring that combine desirable characteristics from both parents [4]. Self-pollination can preserve desirable characteristics and avoid introducing undesirable ones. Plant breeding is based on identifying desirable characteristics in one plant and crossing this plant with another. However, while the desired characteristics then appear in the offspring of this crossing, unwanted characteristics may also be transferred. Removing undesirable characteristics requires subsequent breeding cycles and may take many years [5]. Using conventional plant breeding methods to produce a new crop variety can be a lengthy process, and cannot offer the rapid progress now needed to achieve widespread food security and tackle climate change [6]. Conventional mutagenesis and genetic engineering approaches offer a much faster way to introduce desirable traits into crop plants [7,8].

Transgenic plants are produced artificially using genetic engineering to insert new genes into plant genomes. Most genetically-modified (GM) plants contain new genes transferred through biolistic or Agrobacterium-mediated methods [9]. Well-known transgenic plants include BT crops with improved insect resistance produced by inserting insecticidal genes from a soil bacterium, *Bacillus thuringiensis* [10]. Another example involves introducing a soil bacterium (*Agrobacterium* sp.) gene for CP4 EPSP synthase, an enzyme which confers resistance to the herbicide glyphosate (Roundup) [11].

Because GM plants do not naturally contain these transgenes, their presence triggers biosafety regulations, and the plants are classified as genetically-modified organisms (GMO)s. A common reason for creating transgenic plants is to improve important characteristics, such as shelf life [12], disease resistance [13], quality [14], yield, and tolerance to biotic and abiotic stresses [15]. Other transgenic plants have been created to express proteins for industrial and pharmaceutical applications [16]. With the global population increasing so rapidly, GM plants offer significant economic, health, environmental, and social benefits to farmers, consumers, nations, and regions through higher yields, improved nutritional content, and lower transportation and production costs [17,18]. Manufacturers of GM crops promote these crops as a second ‘green revolution’ that can address worldwide food security problems arising from global warming. In 2019–2020, some 190 million hectares of GM crops were grown across 26 countries. The US, Canada, Australia, Portugal, and Spain account for 46% of GM crops; developing countries such as India, Brazil, and Argentina are also prominent growers of GM crops. The area under GM crops increases every year [19].

The CRISPR/Cas system is a powerful and precise plant-breeding technique for bringing about specific genome modifications that conventional genetic engineering [20,21] cannot achieve. CRISPR/Cas is routinely applied to address problems in crop plants. The technology has the potential to fulfill the early promises of genetic engineering in crops, providing better nutritional value, improved yields, and higher tolerance to biotic and abiotic stresses [22]. The range of new CRISPR/Cas systems and applications now available to improve crops have dramatic implications for food security and food safety [23]. However, despite CRISPR/Cas’s undoubted successes and widespread adoption, there are still concerns about this technology, as with any new technology. CRISPR holds tremendous potential for speeding up the development of plant varieties with superior traits, but the future of this innovative technology hangs on how these crops will be regulated [24,25].

**Figure 1:**
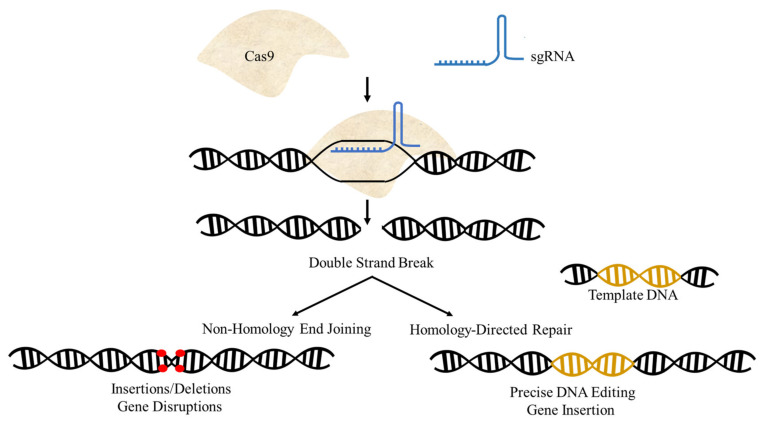
Schematic diagram of CRISPR/Cas9 mechanism. The system consists of Cas9 enzyme and gRNA, which bind with targeted double-stranded DNA to induce DSBs with blunt ends. The break can be repaired by either NHEJ or HDR. HDR requires a template, but NHEJ does not. Both mechanisms result in gene disruptions, deletions, and DNA editing containing the Cas9 gene and gRNA to the host genome at a random location.

Derived from a bacterial defense system, CRISPR/Cas was first used for site-specific rewriting of a eukaryote genomic sequence in 2013 [26,27]. It was declared ‘breakthrough of the year’ by Science magazine in 2015 and its developers were awarded the 2020 Nobel Prize in Chemistry [28]. CRISPR/Cas technology can improve crop genetics in a simple and time-efficient manner [29]. It can precisely cut, remove, replace, or insert specific sequences in the genome [30]. For example (Figure 1), the site-specific endonuclease Cas9 is recruited to the target site by a 20-bp RNA sequence called a guide RNA (gRNA) [31]. Once the gRNA matches its complementary DNA, Cas9 creates a double-strand break (DSB), which can be used to delete, replace, insert, or remove the DNA sequence, depending on whether the repair is achieved by non-homologous end joining (NHEJ) or homology-directed repair (HDR). NHEJ is the preferable mechanism due to its higher efficiency, but it is error-prone. HDR depends on the availability of a donor template for repairing the DSB [32]. Both repair mechanisms have been used with CRISPR to improve crops, but HDR has low efficiency in plants [33]. CRISPR/Cas and associated techniques are frequently used for basic, as well as applied research for improving crop genetics. CRISPR applications are not limited to knocking out plant genes to produce knockout (KO) plants or inserting new genes; they can also be used to introduce site-specific single nucleotide (nt) changes without permanently inserting any transgenes into the host genome [34].

However, although CRISPR/Cas is generally described as precise, off-targets such as unintended deletions, point mutations, inversions, and translocations and unintended effects lead to concern for ecosystem, environmental, and human health-related problems [35]. Further potential concerns arise because the developmental process for most CRISPR-edited plants is similar to that used for conventional GMOs [36]. For example, most CRISPR-edited plants are produced by transferring a DNA cassette.

The process is the same as in GMOs [37]. The Cas9 gene and gRNA cassette produces the functional proteins and gRNA required for creating DSBs and subsequent applications. The scientific community has expressed concern about permanent integration of Cas9 and unintended effects. Although the Cas9 gene can be removed by subsequent crossing of plant offspring, safety-related concerns persist [38]. To overcome these concerns, some scientists have begun using Cas-gRNA complexes known as ribonucleoproteins (RNPs) or in vitro expressed Cas9 mRNA for CRISPR editing in plants. Because RNPs and in vitro transcribed mRNA do not require the use of transgenes, they can often bypass regulations originally designed for GMOs rather than CRISPR-edited plants [39]. Whether edited plants will meet regulations depends on the selection of cargo, delivery system, and ultimate modification.

In plant biotechnology, CRISPR/Cas systems are now routinely used for basic as well as applied research [40,41]. These systems have the potential to fulfill the early promises of genetic engineering, such as plants with higher yield, stronger biotic and abiotic resistance, and better nutritional value [29,41]. CRISPR crops are emerging on world markets, where they provide opportunities to boost food security, but the world is divided over their regulatory oversight. Without a universal, clear, and scalable regulatory system, CRISPR-edited crops may face a similar future to GMOs. If CRISPR-edited crops and derived products are classified as GM crops and regulated the same way, their future cultivation and public acceptance will be low, especially in the European Union (EU) [42]. While CRISPR offers a good degree of precision, the scientific community remains concerned about off-targets [43,44]. Scientists are also concerned about potential environmental impacts and the difficulty of controlling CRISPR-edited crops after release [45]. To date, different countries have regulated genome-edited crops differently as a result of having diverse definitions for genetic modifications and GMOs [46].

## 2. Emerging CRISPR/Cas Systems and Their Applications

### 2.1. CRISPR/Cas Systems

Genome-editing technologies are a revolutionary new development in agriculture [47] with major applications in global crop improvement and food security. CRISPR/Cas has been used to develop new crop varieties indistinguishable from conventionally-bred varieties [48], and crop varieties more resistant to biotic and abiotic stresses that offer better yields and nutritional value. The technology will help farmers and scientists meet the challenge of better food for everyone at affordable prices. Compared with GM crops, CRISPR-edited crops pose minimal risk to ecosystems, human health, and the environment [3]. Barriers to widespread adoption include the risk of unintended genetic modifications, consumer acceptance, and inconsistent regulation of CRISPR-edited crops [49]. The following section discusses different CRISPR/Cas systems and their uses in improving crop genetics. CRISPR/Cas9 is the most widely-used genome editing system, but it is just one of many in the complex world of CRISPR/Cas.

The CRISPR/Cas system consists of two major classes: multi-effector nucleases (Class 1) and single effector nucleases (Class 2) [50]. Both systems are divided into types according to which endonuclease is responsible for DNA/RNA cleavage and mode of action [51]. Class 1 contains Type 1, Type 3, and Type 4 CRISPR/Cas systems. All Class 1 Type 1 systems share common Cas3 loci responsible for helicase activity and target cleavage. Type 1 systems specifically target DNA using multi-subunit ribonucleoprotein (RNP) complex known as ‘Cascade’. Similar to Cas3 in the type 1 system, type 3 systems contain Cas10 loci, responsible for coding RNA recognition and DNA cleavage domains. Type 3 systems target DNA and RNA, and little is known about Type 4 systems [52]. Class 2 systems are only found in bacteria, and consist of Type 2, Type 5, and Type 6, further subdivided into nine subtypes. The best known Class 2 system is Type 2, which contains Cas9, Cas1, and Cas2 proteins. Type 2, the most widely-used genome editing system, requires transactivating CRISPR RNA (tracrRNA) for target cleavage. Type 2 systems target DNA. Type 5 systems are characterized by the presence of Cas12 effector nuclease [53]. Like Cas9, Cas12 targets DNA and requires tracrRNA for DNA cleavage. However, unlike Cas9, which recognizes NGG nucleotide sequences (where N is any nucleotide base; G is guanine) as protospacer adjacent motifs (PAMs), Cas12 recognizes different PAMs and produces staggered ends [54]. Type 6 is the only Class 2 system characterized by Cas13 endonuclease and specifically targets RNA. Cas 13 has been used for RNA editing in plants and can non-specifically cleave RNA, once activated [55].

#### 2.1.1. CRISPR/Cas9 Is the Most Widely-Used Genome Editing System

**Figure 2:**
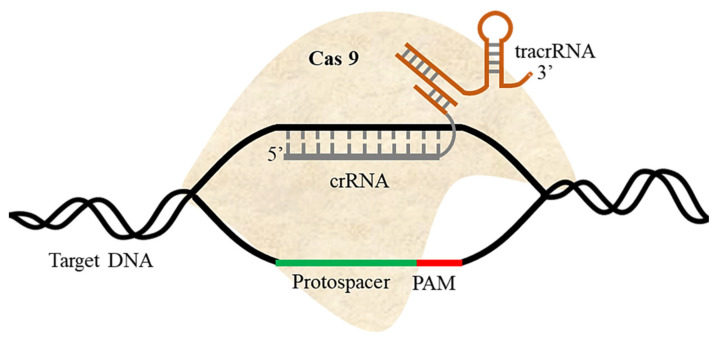
Schematic diagram of CRISPR/Cas9. This system utilizes Cas9 enzyme and two RNAs: crRNA and tracrRNA. On binding with target DNA at the PAM site, it creates DSBs with blunt ends.

Originally identified as a *Streptococcus pyogenes* defense system against invading viruses, CRISPR/Cas9 was the first system used for genome editing in eukaryotes [56]. Cas9 is an endonuclease, which binds with CRISPR RNA (crRNA) and tracrRNA in order to recognize the target DNA [57]. Once the target DNA has been identified by the presence of the appropriate PAM, Cas9 creates a DSB at the target site (Figure 2). Cas9 contains two nuclease domains known as RuvC (a crossover junction endodeoxyribonuclease named for an *E. coli* DNA repair protein) and HNH (named for its characteristic histidine and asparagine residues), each with 1000–1600 amino acids. The gRNA varies from 18–24 nt; truncated gRNA of 17–18 nt shows higher specificity. Some Cas9 variants recognize PAMs other than NGG, increasing the range of potential target sites. TracrRNA and crRNA are fabricated as single gRNA in an engineered system used for genome editing in eukaryotes. Changes in gRNA sequence enable Cas9 nuclease systems to target any genome sequence where a suitable PAM is present. The system’s major limitations are the large size of Cas9 and the risk of off-target impacts [58,59,60,61].

#### 2.1.2. CRISPR/Cas12 Has Advantages over Cas9

**Figure 3:**
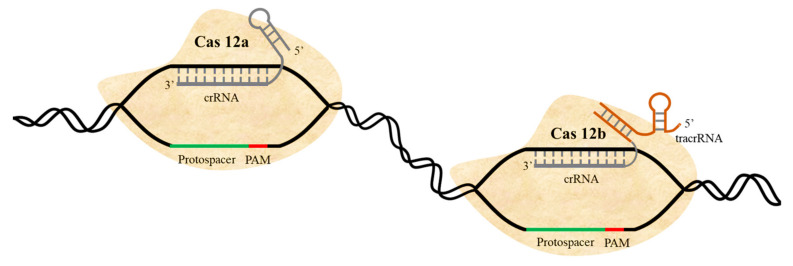
Schematic diagram of CRISPR/Cas12a and 12b. Both bind with target DNA upstream of PAM to induce DSBs with staggered ends. Cas12a utilizes crRNA. Cas12b utilizes crRNA and tracrRNA.

Like Cas9, the CRISPR/Cas12 system is a Class 2 system that creates programmed DSBs. It has been used as an alternative system for genome editing in plants, with comparable efficiency to Cas9 [62]. Cas12 requires a shorter crRNA than Cas9 does to create DSBs. Cas12 is smaller than Cas9 at around 1200 amino acids (aa) [63]. (Cas12b from *Alicyclobacillus acidoterrestris* and *Alicyclobacillus acidiphilus* are 1129 aa) and requires a 22-nt gRNA spacer for maximum efficiency. Cas9 recognizes a G-rich PAM (NGG) at the 3′ end, but Cas12 recognizes a T-rich PAM (5′-TTN for FnCas12a) at the 5′ end of the guide sequence (Figure 3). Cas12a, which is based on crRNA (while Cas12b uses an additional small tracrRNA as well), reportedly [64] cleaves single-strand DNA (ssDNA), as well as double-strand DNA (dsDNA), and once activated, it cleaves ssDNA indiscriminately. Cas12a processes its pre-crRNA into mature crRNA without requirement of tracrRNA. Whereas Cas9 creates blunt ends at the cleavage site, Cas12 produces a staggered end. Cas12 has been used to develop a diagnostic method called DETECTR for ultrasensitive detection of infectious diseases. DETECTR has been used to detect various pathogens with high efficiency, accuracy, and speed [65]. In addition, loop mediated isothermal amplification (LAMP)-coupled Cas12a has been recently developed to accurately detect plant viruses [66]. The superior features of Cas12 compared to Cas9—smaller size, recognition of T-rich PAM, and production of staggered ends—make Cas12 a viable and powerful alternative tool for genome editing in crop plants [63,67].

#### 2.1.3. CasX Is Smaller Than Cas12

**Figure 4:**
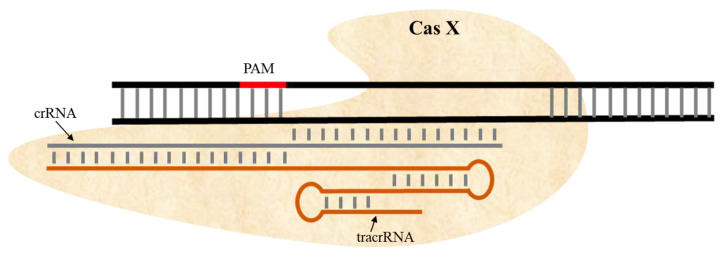
Schematic diagram of CRISPR/CasX. CasX is a dual-guided RNA enzyme utilizing crRNA and tracrRNA. It binds and cleaves dsDNA adjacent to an appropriate PAM site.

The latest addition to Class 2 CRISPR/Cas systems for genome editing is CasX. CasX was identified by multigenomic analysis of microbial DNA from groundwater [68]. It prevents bacterial transformation when gRNA is targeted to plasmid DNA. CasX has little in common with Cas9 or Cas12 other than RuvC, indicating that CasX has a distinct function from other Cas enzymes [69]. Moreover, the RuvC nuclease domain of CasX shows 16% similarity with the RuvC domain of Cas9 or Cas12a. CasX has been shown to work as an RNA-guided endonuclease to cleave dsDNA and produce staggered ends. CasX recognizes 5′-TTCN. With just 980 amino acids, it is smaller than either Cas9 or Cas12a (Figure 4). CasX appears to be a hybrid enzyme with some elements from both Cas9 and Cas12a but with novel RNA folds and protein domain. Its small size makes it the best choice for genome editing compared with Cas9 and Cas12a [70,71,72].

#### 2.1.4. Cas14a Only Binds and Cleaves ssDNA

**Figure 5:**
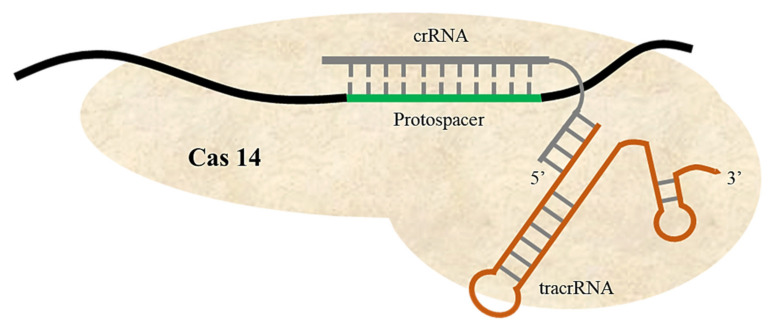
Schematic diagram of CRISPR/Cas14. This system utilizes Cas14 enzyme with two RNAs: crRNA and tracrRNA. It binds only ssDNA, and cuts without requiring a PAM site for recognition.

In 2018, researchers seeking a simple and efficient CRISPR/Cas system used metagenome analysis of microbial genomes to reveal new, uncharacterized CRISPR/Cas14 systems [73]. CRISPR/Cas14 systems have only been characterized in archaea, i.e., not in bacteria. Cas14a is much smaller than other CRISPR/Cas nucleases, including Cas9, Cas12a, and CasX [74]. It has approximately 400–700 amino acids (40–70kD), making it roughly half the size of Cas9. The Cas14 gene has 24 variants, which are grouped into three subgroups: Cas14a, Cas14b, and Cas14c [75]. All Cas14 proteins are characterized by a conserved RuvC domain. Compared with other Cas systems for DNA targeting, Cas14 specifically binds and cleaves ssDNA (Figure 5). Although Cas14 specificity is programmed by guided RNA, it does not require a PAM sequence—unlike other DNA targeting CRISPR/Cas systems [76]. The ability to target ssDNA makes Cas14 systems a powerful tool to defend crops against ssDNA viruses and genetic elements that replicate through ssDNA intermediate structures [77]. Cas14 is also suitable for developing tools for diagnosing viral and bacterial infections and detecting cancerous cells [76].

#### 2.1.5. CRISPR/Cas13 Is an RNA Editing System

Cas13 is a Class 2 type VI system, which specifically targets RNA. CRISPR/Cas13 is further divided into VI-A, B, C, and D subtypes [78]. Cas13 is characterized by the presence of higher eukaryote and prokaryote nucleotide-binding (HEPN) domains [79]. It ranges in size from 900 to 1300 amino acids and requires a gRNA spacer of 22–30 nt to recognize target RNA sequences, followed by a protospacer flanking sequence (PFS). For example, LshCas13a recognizes a 22–28 nt target sequence with a PFS of H, where H denotes a non-G base (i.e., A, U, C) [80]. In prokaryotic cells, HEPN activation by target RNA is followed by cleavage of non-target RNA, indicating a role in programmed cell death. However, non-specific cleavage of RNA (collateral cleavage) has not been observed in genome editing experiments in eukaryote cells. Like Cas12a, Cas13a processes pre-crRNA to a mature crRNA. This ability to process pre-crRNA can be used to manipulate multiple gRNAs for multiplex RNA editing with Cas13a [81]. CRISPR/Cas13 has been used for RNA tracking [82], RNA knockdown [83], and viral detection.

Starting by fusing CRISPR/dCas13 with an ADAR2 (a member of adenosine deaminase family acting on RNA) domain, scientists from MIT McGovern Institute for Brain Research developed new tools for creating temporary genome changes by editing RNA bases. These are referred to as RESCUE (RNA editing for specific C to U exchange) [84,85] and REPAIR (RNA editing for Programmable A to I (G) replacement) [84]. While CRISPR/Cas 9 and CRISPR/Cas12 permanently change DNA, CRISPR/Cas13 can make temporary and reversible changes at transcriptional level [86]. This avoids potential risks and ethical issues associated with DNA-based genome editing. CRISPR/Cas13 has also been widely used for diagnostic purposes. A system known as Specific High-sensitivity Enzymatic Reporter un-LOCKing (SHERLOCK) has been developed for detecting RNA with attomole efficiency [83].

### 2.2. Innovations in CRISPR/Cas Systems

After CRISPR/Cas genome editing was first reported in 2012, it become a favorite genome engineering tool in both plants and animals [87]. CRISPR/Cas systems have enabled enormous advances towards solving key problems in agricultural [21] and medical sciences [88]. CRISPR-based genome editing has been used for gene knockout [89,90], gene replacement [91], activation/repression [92,93], base editing [94], gene insertion [95], and to search for and characterize new genes. Because off-target impacts are a major limitation on food, agriculture, and clinical applications of CRISPR/Cas [96], scientists are continuing to seek ways to improve CRISPR/Cas systems. This section discusses examples of CRISPR/Cas system innovation in agriculture and biological sciences.

#### 2.2.1. Deactivated Cas Nuclease Systems Can Bind DNA but Not Cleave It

By mutating the nuclease domains of Cas enzymes, such as Cas9 and Cas12a, scientists have produced deactivated nucleases (dCas9, dCas12a, dCas13a) that can bind DNA but not cleave it. DCas12a and dCas9 can be fused with transcriptional activators or inhibitors to recruit them to target genes [97,98]. dCas9 and dCas12a can also be fused with epigenetic modifiers or other effector domains for genome imaging [99,100]. dCas systems can readily be modified for multiplex genome editing [101].

#### 2.2.2. Base Editing with CRISPR/Cas Systems

**Figure 6:**
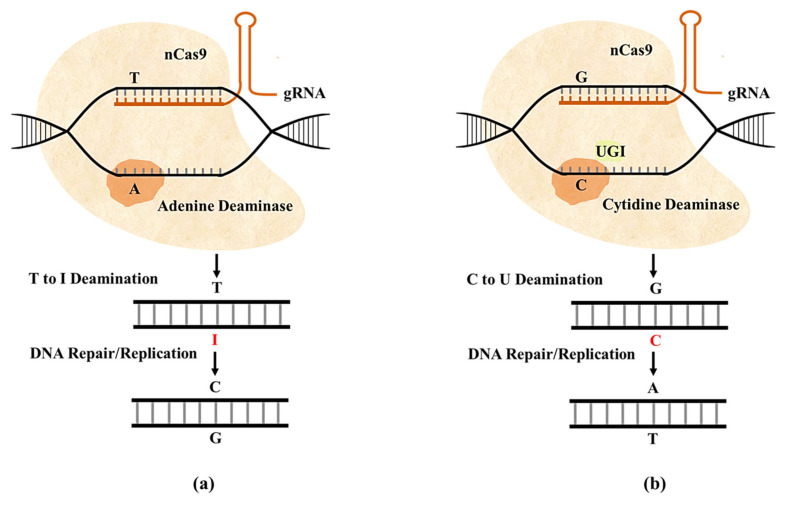
Schematic diagram of base editing with nickase Cas9 (nCas9). (**a**) ABE system uses nCas9 and adenine deaminase to catalyze transformation of adenine into guanine. ABE deaminates adenine to inosine (I), thus converting T-A to T-I. Repair machinery recognizes I as G and repair T-I as C-G; (**b**) CBE system utilizes nCas9 and cytidine deaminase to catalyze transformation of cytosine to uridine. Uracil glycosylase inhibitor (UGI) prevents U:G mismatch from being repaired back to C:G, and U is ultimately transformed into T.

Coupling the dCas9 system with either cytosine deaminase (cytidine base editor (CBE)) or adenosine deaminase (adenine base editor (ABE)) enables introduction or correction of point mutations in a genome [102]. By recruiting base editors to the target site using gRNA, these systems can replace C-G with T-A or A-T with G-C without cleaving DNA (Figure 6) [101]. As base editing is specific and does not involve random cleavage, outcomes are predictable. However, due to the restricted base editing window, this process cannot be applied to all target sites in a genome [94,103].

#### 2.2.3. RNA Editing Systems

Cas13 systems that exclusively target RNA are another useful addition to the CRISPR/Cas toolbox. Cas13 is a Class 2 type VI CRISPR/Cas system containing a single effector. It is the first-known Class 2 system with a single effector nuclease that can bind and specifically cleave ssRNA, catalyzed by HEPN domains [104]. Like Cas12, Cas13a processes pre-crRNA to mature crRNA and can therefore be used for multiplex RNA editing. In multiplex RNA editing, pre-crRNA processing ability of Cas13, can be used to target multiple RNA targets by processing multiple crRNA from a single array. Unlike Cas9 and Cas12, which recognize PAM sequences adjacent to a target sequence, Cas13a recognizes a PFS followed by a target sequence [105]. However, while LshCas13a from *Leptotrichia shahii* requires a PFS for target identification [67], LwaCas13a (from *Leptotrichia wadei*) and PsPCas13b (from *Prevotella* spp.) do not appear to require a PFS for target identification [83,106]. Once Cas13a is activated in prokaryotic cells, it cleaves non-target RNA, indicating a role in programmed cell death [107]. However, such collateral cleavage has not been observed in eukaryotic cells [108]. Cas13a’s ability to non-specifically cleave RNA has enabled development of a SHERLOCK diagnostic method for detecting specific RNA sequences. Base-editing systems, such as REPAIR and RESCUE, utilize dCas13 fused with ADAR2 adenine or cytidine deaminase domains [109]. Cas13 and dCas13 have also been used for RNA knockdown, splicing regulation, RNA labeling, and detection of viral materials. RNA editing is more efficient and specific than DNA editing. A further advantage is that temporary and reversible editing with CRISPR/Cas13 avoids the potential risks and ethical issues associated with permanent genome editing. RNA editing with CRISPR/Cas13 has opened up a new era for diagnostics, clinical, and therapeutic treatments [110].

#### 2.2.4. Prime Editing: A New CRISPR/Cas Development for Precise Editing

**Figure 7:**
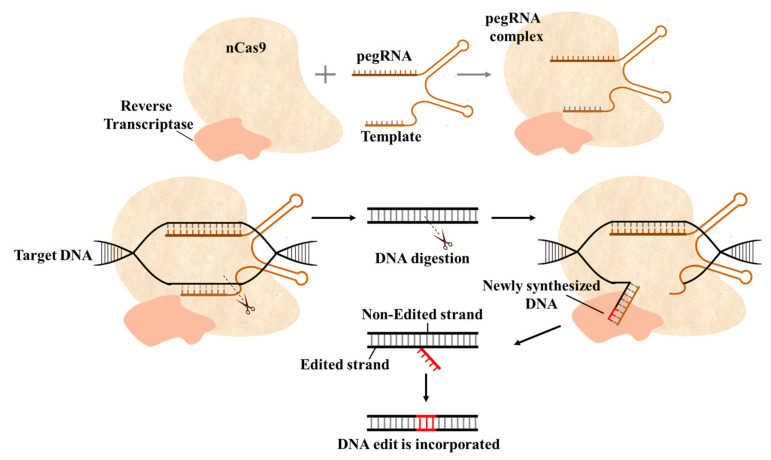
Schematic diagram of prime editing, which involves fusing nCas9 with reverse transcriptase and a prime editing guide RNA (pegRNA). Prime editing systems edit DNA without causing DSBs, and the reverse transcriptase can accomplish various transitions, insertions, and deletions.

Prime editing is a recent development in CRISPR/Cas systems. It can be used to perform all 12 types of base substitutions [111], targeted insertions [112], deletions without creating DSBs, and the provision of donor template [113,114]. In prime editing, nCas9 is fused with reverse transcriptase (RT) and a prime editing guide RNA (pegRNA), a modified gRNA with extensions to encode primer binding site (PBS), RT-template for targeted modifications in the target sequence and a canonical gRNA for the second nick (Figure 7). After nCas9 cleaves the target site, reverse transcriptase uses pegRNA to introduce new genetic information at the target site. Prime editing has the potential to expand the scope of genome editing, especially in therapeutic applications. Although it has been suggested that prime editing could, in theory, correct 89% of known genetic mutations, such systems still need further improvement before they can be used for therapeutic purposes [113,115]. Prime editing holds an extraordinary potential to introduce precise modifications in crop genomes for functional genomics, stress tolerance, yield improvement, and disease resistance. Prime editing has been demonstrated for targeted genome editing in different plants, such as wheat, rice [116], and *Arabidopsis* [117].

## 3. Classification of Genome Editing Modifications

**Figure 8:**
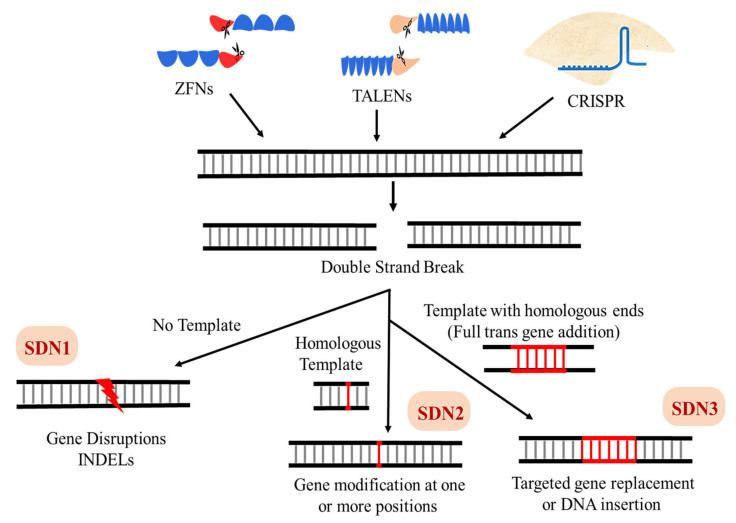
Schematic diagram of SDN1, SDN2, and SDN3. Nucleases such as ZFNs, TALENs, and CRISPR/Cas9 bind with target DNA to cause DSBs that are repaired by two different mechanisms. SDN1 does not need a template and results in gene disruptions through indels (small insertions or deletions of bases). SDN2 utilizes a homologous template and results in gene correction or modification at one or more positions. SDN3 requires a full gene as a template, and leads to gene replacement or foreign DNA insertion.

Both conventional mutagenesis and genetic engineering are based on stable but random genetic modification. Random modifications may cause undesired alterations in the genome, potentially disrupting genes, altering cis-regulatory elements such as promoters, silencers, or enhancer sequences, or creating toxic new proteins. GMOs created through random changes often require lengthy screening and selection, and typically trigger strict regulatory processes before they can be released to market [118]. New technologies such as site-directed nucleases (SDNs) have emerged as sophisticated tools to introduce desired traits into plants. SDN techniques include zinc finger nucleases (ZFNs), transcription-activator like (TAL) effector nucleases (TALENs), and CRISPR/Cas technology (Figure 8). SDNs make precise modifications at predefined locations in a genome, thereby reducing the likelihood of random mutagenesis. SDNs provide unprecedented control over precise genome modifications, with greater cost-effectiveness and efficiency than plant breeding and genetic engineering. Plant varieties created using SDNs do not contain exogenous genetic material and could have resulted from natural processes [119,120,121]. SDN applications may result in three types of plants:

- Plants with new genetic element;
- Plants with point mutations in existing DNA but no new DNA;
- Plants with no genome modification.

SDNs could potentially help developing countries to improve crops along with food security. Depending on genome modification and DSB repair outcomes, SDNs are classified as SDN1, SDN2, and SDN3 (Figure 8) [122]. This section discusses those modifications in detail.

### 3.1. SDN1 Systems

Applications of SDN1 systems involve introducing a precise point mutation that leads to gene disruption, KO, or silencing. With DSB repair in SDN1, a repair template is not needed to introduce the desired modifications. Scientists are currently debating the extent to which off-targets in SDN1 should be studied. SDN1 outcomes are indistinguishable from natural mutations [122,123].

### 3.2. SDN2 Systems

SDN2 systems can precisely modify a single base or a small number of bases at a DSB using a repair template. The outcomes are indistinguishable from natural mutations, and theoretically achievable through conventional plant breeding methods. The repair template contains the desired base(s) to be copied into the DSB. SDN2 is not suitable for introducing a larger template into DSB because it involves a microhomology end-joining (MHEJ) pathway that employs homologies from six to 20 nt to copy a template. MHEJ has some features in common with HDR but differs overall [124].

### 3.3. SDN3 Systems

SDN3 applications involve a repair template with a perfectly homologous region of 500 or more base pairs (bps) on both upstream and downstream ends of DSB. SDN3 is mostly used to introduce new sequences at DSB, and it achieves outcomes comparable to transgenic or cisgenic methods. Applications of SDN3 are inefficient and complicated in plants due to low efficiency of HDR in plants [124,125,126].

## 4. CRISPR/Cas Reagents and Their Cargos

The rapid rise in CRISPR-edited crops in recent years has raised concerns about regulatory oversights. As a consequence, researchers have focused on developing CRISPR-edited crops that are comparable to conventionally-bred crops, with no transgenes and minimal off-targets [127]. Two particular concerns are off-targets and the presence of genome-editing reagents in final products. Reagent selection, dose, duration of exposure, and delivery method significantly impact the elimination of transgenes and off-target in the final product. For example, using Agrobacterium to deliver CRISPR reagents (Cas9 and gRNA genes) typically leads to these genes being randomly integrated into the plant genome, and with the resulting plants being similar to transgenic plants [128]. Permanent integration of Cas9 and gRNA genes can also lead to continuous expression, increasing the possibility of off-targets. Although transient expression in protoplast-mediated transformations can reduce off-targets, protoplast transformation does not work in all crops. Direct introduction of Cas9 and gRNA into a cell is a possible alternative to transient approaches for plant-mediated genome editing [129]. The two prerequisites for CRISPR/Cas-mediated genome editing are a gRNA for site-specific targeting and a nuclease (e.g., Cas9) for cleavage at the target site. Although a 20-bp sgRNA recruits Cas9 to the target site, Cas9 can tolerate mismatches within sgRNA and non-selective cleavage may lead to off-targets. SgRNA specificity is the first step towards limiting binding and cleavage at non-target sites and thereby reducing off-targets [130]. Several in-silico tools are available to evaluate the on– and off-target specificities of sgRNAs [131]. Once a specific sgRNA has been designed and an appropriate Cas9 selected, they can be delivered as DNA, RNA, or RNPs. Guide RNA specificity, CRISPR reagent choice, and selected delivery method collectively influence outcomes, likelihood of regulatory oversight, and public perceptions of CRISPR-edited plants. This section discusses CRISPR reagents and delivery methods in various applications.

### 4.1. CRISPR Reagents

Reagent selection in CRISPR-mediated plant genome editing can facilitate commercial applications, influence regulatory processes, control off-target numbers in the final product, and affect public acceptance of edited plants and products. CRISPR reagents (sgRNA and Cas9) for plant genome editing can take the form of DNA, RNA, or RNPs [127,132].

#### 4.1.1. Plasmids

Plasmids are common CRISPR reagents for plant genome editing. They offer advantages, such as multiplex genome editing—multiple gRNAs expressed in a single plasmid, stability and prolonged expression. Plasmid-based expression of Cas9 requires a promoter and a nuclear localization sequence (NLS) [133]. However, plasmid-based delivery of CRISPR reagents also has its challenges, including the risk of permanent and random integration in the genome. Plasmid size can sometimes be a problem. For example, multiplex genome editing, where several sgRNAs are expressed from an individual promoter, requires large plasmids, which make transfection—the process by which DNA or RNA is introduced into cells—less efficient. Permanent and random integration of plasmids produce genome-edited plants similar to transgenic plants, which are subject to strict GMO regulations. Permanent integration also increases off-target impacts [134].

#### 4.1.2. Messenger RNA

Using mRNA as a CRISPR reagent offers an alternative, transient method of genome editing with several advantages. For example, mRNA can be directly translated in the cytoplasm, and does not need nuclear localization for transcription. Using mRNA also makes permanent integration less likely, thereby reducing off-targets and the risk of insertion mutagenesis. Disadvantages of mRNA genome editing include poor stability and reduced efficiency [133,135].

#### 4.1.3. Ribonucleoproteins (RNPs)

RNP systems offer the fastest and most straightforward CRISPR-based genome editing. RNP delivery of CRISPR/Cas9 systems does not require Cas9 to be transcribed or translated inside the cell. RNPs minimize the risk of poor stability with mRNA and the risk of permanent integration with plasmids [133]. Compared to plasmids and mRNA, RNP systems are faster acting and more transient, with reduced off-targets, toxicity, and immune response [134]. RNPs can form complexes with mRNA formed from sgRNA and move across nuclear membranes. They hold great potential for HDR-mediated genome editing in plants and the generation of transgene-free CRISPR-edited plants [133]. However, RNPs have their own limitations. For example, RNP applications are limited by the large size of Cas9 and the presence of opposing charges on Cas9 (positive) and sgRNA (negative) [135]. Cost and purity are additional challenges for using RNP as CRISPR reagents. Despite these disadvantages, RNPs have already been used to edit plant genomes [136,137].

### 4.2. Delivery Methods in Plants

The methods used to deliver CRISPR reagents are crucial for producing transgene-free, genome-edited plants that can bypass GM regulations and quickly improve food security. Although DNA is the most commonly-used reagent in CRISPR-based genome editing, permanent integration of plasmid DNA can increase off-targets and produce genome-edited plants similar to transgenic plants, limiting commercial applications and public acceptability.

Mendelian segregation is a way to obtain transgene-free plants with better public acceptance. Using Mendelian segregation to remove transgenes from genome-edited plants produces null segregants, plants that retain only the desired genome change. Suicidal genes can prompt programmed self-elimination of transgenes; for example, bacterial BARNASE and rice CMS2 genes have been used to obtain rice null segregants. However, this approach does not work for asexual propagation of plants [21,138]. Transient expression of CRISPR reagents is another way to obtain transgene-free plants. Andersson et al. and Lin et al., achieved transient expression of Cas9 in the protoplast of several plant species (potato, rice (*Oryza sativa*), *Brassica oleracea*, and *Nicotiana benthamiana*) [139,140]. However, protoplast transformation has the disadvantage that some plants cannot be regenerated from protoplast. Transgene-free plants can also be produced by Agrobacterium-mediated transient transformation. However, while transient transformation has had successful applications, degraded DNA fragments can still become integrated into the host genome [141].

Using RNPs in DNA-free CRISPR-based genome editing to produce transgene-free plants could improve food security in developing countries. Because RNPs do not contain DNA, permanent transgene integration can be avoided. Several studies have reported successful RNP applications in plants [142,143].

Methods for delivering CRISPR reagents in plants are characterized as direct (physical and chemical) or indirect (Agrobacterium and viral). This section discusses delivery methods in plants.

#### 4.2.1. Direct Delivery Methods

Direct methods for CRISPR reagent delivery in plants include physical (biolistics) and chemical (polyethylene glycol (PEG) mediated) methods, which together comprise the most commonly-used direct methods in plants. Both have been successfully used for CRISPR applications in plants. However, both have limitations [144]. Biolistics, one of the most common physical methods used in plants, uses high-speed bombardment of cells to deliver multiple constructs with reasonable efficiency [129]. However, this system is limited by its tendency to permanently integrate multiple constructs. Scientists have used biolistics to deliver CRISPR reagents in soybeans [145], wheat [146], rice, and maize [147]. PEG– and electroporation-mediated transformations of protoplast have been used for direct delivery of CRISPR reagents, and can deliver DNA, mRNA, or RNP. However, although protoplast transformation has been used to demonstrate CRISPR applications in different plant species [139,148], regeneration from protoplast is not possible for all plant species. Other direct methods, such as nanoparticles, cell-penetrating peptides (CPPs), and ‘whiskers’ (hard, cleavable microfibrils able to puncture the cell wall minutely for transfer of DNA), have not been applied to CRISPR-based genome editing in plants.

#### 4.2.2. Indirect Delivery Methods

Indirect methods involve Agrobacterium– and plant virus-mediated delivery of plasmids as CRISPR reagents [129]. Agrobacterium-mediated delivery is one of the cheapest and most convenient methods for delivering CRISPR reagents in plants. Agrobacterium has been successfully used for CRISPR applications in model and crop species [149,150], and to deliver single and multiplex CRISPR constructs. However, like all other methods, this method has disadvantages. For example, Agrobacterium-mediated delivery of CRISPR reagent always results in transgenic plants and cannot deliver small DNA fragments, RNA, or proteins [129]. Moreover, it is genotype-dependent. In viral systems, tobacco rattle virus (TRV) has been suggested as an efficient system for editing plant genomes. Because the TRV genome consists of ssRNA, in-vitro transcribed RNA may also work for infection and virus-induced gene silencing. Because TRV does not integrate its RNA into the plant genome, it could potentially be an efficient delivery system for producing transgene-free, CRISPR-edited plants [151,152]. Single-stranded DNA geminiviruses have been used as delivery agents through plasmids [153]. Such viruses may soon be employed as efficient, alternative systems for CRISPR/Cas delivery.

## 5. Potential Concerns Associated with CRISPR Crops

Since its first demonstration in plant genome editing in 2013, the rapidly expanding CRISPR/Cas toolbox has given researchers unprecedented control over precise genome modifications for improved plant genetics. CRISPR/Cas-based platforms such as prime editing [154], directed evolution [155], and base editing have been developed for targeted genome editing but their full potential in plant biotechnology is yet to be achieved [156]. CRISPR could potentially address most of the problems associated with genetic modification of crop plants, including random insertion, antibiotic resistance genes, and insertional mutagenesis (integration of exogenous DNA sequences within the host genome). Transgene-free CRISPR-edited crops, particularly those produced using SDN1 and SDN2 systems, are especially important in the context of global climate change and food security challenges [157]. Moreover, multiplex genome editing and stacking multiple alleles into a single specific locus with CRISPR/Cas will speed up plant genetic improvement with multiple traits. As a result of recent CRISPR/Cas developments, plant biotechnology has entered a new era of precisely customized crops not attainable using traditional genetic engineering. The success and future potential of CRISPR/Cas in agriculture has initiated an international debate over the likely impacts associated with CRISPR crops, how these crops differ from GMOs, and how CRISPR crops should be regulated.

In addition to its role in revolutionizing scientific research and agricultural applications, CRISPR has emerged as an economic game-changer [36]. CRISPR/Cas has significantly lowered the cost of producing genome-edited crop plants and holds enormous potential to continue reshaping the future of agriculture with precisely edited crops. However, although CRISPR crops are emerging on world markets, the scientific community is divided over the potential risks associated with CRISPR-edited crops. CRISPR’s outcomes are not always predictable and unintended genome modifications may occur due to non-specific binding of sgRNA. Moreover, most CRISPR/Cas systems achieve their precise genome modifications using the same transformation and re-generation protocols as GM methods. This section discusses potential risks associated with CRISPR crops that may trigger regulation of such crops.

Although CRISPR crops are being grown and developed worldwide, this trend has been accompanied by much debate about legal, ethical, and policy issues associated with these crops [127]. This has typically focused on the relative precision and specificity of different CRISPR techniques, the frequency and nature of off-targets, the nature of risk assessment methods for these crops, and whether existing GMO regulations should be applied to CRISPR-edited crops [122].

Many scientists say modifications made using CRISPR are no different from natural or conventional breeding—and therefore CRISPR-edited varieties should not be subject to existing GMO regulations. However, there is international debate about whether these new crops should be assessed under conventional GMO regulations or allowed to reach market without regulation. For example, the US and EU assess CRISPR-edited crop plants under very different regulatory frameworks. Although many countries have clarified their regulatory frameworks to exclude CRISPR crops produced using SDN1, SDN2, and SDN3 systems [127], most are still based on existing GMO regulations.

The international community has been considering two important issues in relation to CRISPR-edited crops. Firstly, is it possible to exclude certain CRISPR-edited crops from regulatory oversight? This is especially important for SDN1 and SDN2 modifications, which are transgene-free and indistinguishable from conventional crops. Secondly, for CRISPR-edited crops to be regulated in specific countries, what safety data would be required? The amount of safety data required will affect the overall cost of regulation, an important factor to consider when bringing new CRISPR plants to market. Regulatory requirements influence decisions about where to invest in CRISPR-edited crops. Some regulatory authorities have been using a case-by-case approach to decide whether CRISPR-edited products are GMOs or not. Developers and investors need to consult regulatory agencies before making any significant investment [158].

Engineered CRISPR/Cas systems rely on two main components: a Cas endonuclease and a gRNA [159]. For genome-editing applications, CRISPR relies on the specific binding sites and nuclease activities of Cas nucleases (mostly Cas9, Cas12, and Cas13) directed by gRNAs. Regardless of application, Cas nucleases and sgRNAs can take the form of plasmid DNA, in-vitro transcribed mRNA, or RNPs. Delivering these reagents involves Agrobacterium, biolistic, or viral-mediated methods, or protoplast-mediated transformation. Selection of reagents and delivery methods has a major influence on end products and consequently on the regulatory requirements for final products [127]. The following section discusses various reagents and delivery methods together with their downstream effects, such as off-target impacts, regulating triggers, and product acceptance.

### 5.1. Selection of Reagents for Creating Genome-Edited Crop Plants

CRISPR/Cas applications in plants are dominated by the use of plasmids to deliver CRISPR reagents. Various studies have already demonstrated the potential of plasmid-based reagents. However, using plasmids for CRISPR reagents requires the construction of expression cassettes and vectors, which is often sophisticated and laborious work. For multiplex genome editing, construction may become even more complex and necessitate professional skills. Plasmid-based systems may lead to permanent integration of recombinant DNA in the host plant genome and generate transgenic plants. Permanent integration would result in continuous expression of Cas and gRNA, thereby increasing the risk of off-target impacts, triggering regulatory oversight of the end products, and limiting commercial application of edited crops. Although transgenes can largely be removed by traditional plant-breeding processes, such as selfing and crossing, unintended DNA fragments may remain in the genome. Segregation methods can also deal with transgenes, but not in plants that reproduce asexually. Transgenes can be marked using fluorescent cassettes to monitor the presence of transgene, thus allowing selection of transgene-free genome-edited plants. Suicide genes, such as CMS2 and BARNASE, can be used to kill transgene-containing pollens and embryos.

Transient gene expression is an alternative method for producing transgene-free, genome-edited plants [21,133,138]. Its first application was to deliver CRISPR reagents into immature wheat embryos using particle bombardment; regenerated plants were selected without antibiotics. Transient gene expression has also been achieved by protoplast transformation in potato and tobacco plants [139,160,161]. Agrobacterium-mediated transient transformation of CRISPR constructs has been reported in tobacco [161]. However, even transient transformation can cause unintended DNA to become integrated into the host genome. Further, protoplast regeneration is not available for all plant species. The limitations of plasmid reagents for transient transformation can be overcome using in-vitro transcribed mRNA of Cas9 and sgRNAs to generate transgene-free plants [129]. For example, Zhang and his team used particle bombardment to deliver in-vitro transcripts of Cas9 and sgRNA into immature wheat embryos [162]. However, although using mRNA reduces off-targets, editing efficiency is much lower than with DNA expression systems, possibly due to RNA instability. Some researchers have used Cas9-sgRNA RNPs to avoid the limitations of DNA– and mRNA-based CRISPR/Cas reagents. Cas9-sgRNA RNPs are more efficient than DNA-based editing systems, and produce fewer off-target impacts. RNPs also have the advantage of not requiring the transcription and translation machinery needed for plasmid– and mRNA-based systems [163]. In 2015, Woo et al. demonstrated PEG-mediated RNP transformations in rice, tobacco, lettuce, and *Arabidopsis* protoplasts. They detected mutations in lettuce and *Arabidopsis* with no off-targets [39]. CRISPR/Cas9 RNP has also been used for genome editing in grapes (*Vitis vinifera*), apple, and potato protoplast [139,164]. Kim et al. used Cas12 RNPs with in soybean and tobacco [137]. RNPs have been delivered by particle bombardment in maize and wheat [146,163]. Collectively, these studies demonstrate that RNP delivery by particle bombardment is a valuable method for generating transgene-free, CRISPR- edited plants.

### 5.2. Selection of Delivery Method

The development of effective and universal methods for delivering CRISPR/Cas reagents into plant cells is a challenging task. The most commonly-used method, Agrobacterium-mediated delivery, has serious limitations, as does the second most common method, biolistics. Agrobacterium-mediated delivery can only be used for DNA, it cannot achieve transgene integration, and its transformation efficiency varies greatly with recipient genotype. Biolistics delivery of DNA reagents inevitably leads to integration at random sites in the host genome. Both methods generate transgenic plants, thereby triggering regulatory oversight for such crops. Moreover, both methods rely on lengthy tissue culture procedures [129]. To reduce the risk of permanent integration, in-vitro transcribed Cas nuclease and sgRNA can be co-delivered by a biolistics method. Because biolistics delivery of RNPs can generate CRISPR-edited plants without the need to use exogenous DNA, and RNPs present transiently, plants edited in this way would be transgene-free [146]. RNPs also enable multiplex genome editing through delivery of multiple sgRNAs [29]. However, although proof-of-concept studies have demonstrated the potential of RNPs in CRISPR applications, RNP procedures have their own limitations, including problems in protoplast regeneration, and the costly and laborious identification processes required for CRISPR-edited plants [129]. Protoplast regeneration can be accelerated by using morphogenetic regulators.

A promising alternative approach that avoids tissue culture involves using plant viruses to obtain CRISPR-edited plants [151]. For example, positive-sense ssRNA viruses such as TRV, tobacco mosaic virus (TMV) and barley stripe mosaic virus (BSMV) have been used to deliver genome-editing reagents in plants [151,165]. Although viruses have limited cargo capacity, RNA viruses do not integrate their genome into a host plant’s genome and can therefore be used to generate transgene-free genome-edited plants. Several studies have used plant viruses such as TMV, TRV, and ssDNA geminiviruses for CRISPR editing in plants [151,152,153]. A relatively simple and inexpensive method called lipofection was recently used to deliver RNP into tobacco protoplast, with 6% editing efficiency. For this process, mixing RNPs with cationic lipids produces positively-charged liposomes that can merge with negatively-charged protoplast membranes. Lipofection produces transgene-free plants without triggering regulatory hurdles [166]. Electroporation and electrotransfection processes have been used for genome editing in single-cell green alga (*Chlamydomonas reinhardtii*) and cabbage protoplast [167,168]. These methods could be further improved to achieve DNA-free genome editing in plants.

### 5.3. Off-Target Impacts

Specificity is essential in CRISPR genome editing for human therapies, commercial crops, and animal production. However, concerns associated with genome-edited animals or human therapeutics may not apply to plant crops to the same extent. Although CRISPR/Cas is theoretically a precise editing tool, its tolerance of a certain degree of mismatches between sgRNA and target DNA can lead to off-target effects. Off-target impacts are particularly important in human therapeutics because they can directly affect treatment outcomes and may lead to genotoxicity, cytotoxicity, or chromosomal rearrangements. Off-targets in mammalian cells can be greatly reduced using truncated sgRNA around 17–18 nt long [169]. In plants, by contrast, off-targets generally remain a technical challenge because researchers can use processes such as regeneration and segregation to help them detect, evaluate, and restrict unwanted mutations and phenotypes. Any unwanted or off-target mutations that negatively affect plants can be regulated in subsequent generations. Most off-target impacts reported to date have been in human cell research rather than plant cells [170,171,172]. Off-target mutations in human cancer cells have been attributed to sgRNA mismatching of dysfunctional repair mechanisms [173]. Similar off-targets have been observed in farm animals, including pigs and cattle [174,175], and non-specific editing has been detected in genome-edited mice [176]. Other studies have shown low or no detectable off-targets in genome-edited animals, possibly because it can be difficult to distinguish between off-targets and natural variations. In contrast to animals and human cells, whole-genome sequencing for off-target effects has revealed limited off-targets in *Arabidopsis*, rice, and tomato. Off-targets in CRISPR-mediated editing of plant genomes have been linked to various factors, including sgRNA specificity, dose-dependent Cas nuclease expression, and reagent selection [127]. Mismatches in the distal region of sgRNA may also lead to off-targets. Various software packages are available to evaluate gRNA off-targeting during the design process and minimize unwanted mutations. For example, CGAT [177], CRISPR-P [178], and CHOPCHOP [179] software packages are useful for predicting off-targets in plant genomes. CRISPR nickase systems can also reduce off-target impacts in plant and human cells [180,181]. The dose and time-dependent nature of CRISPR reagents may increase on-target efficiency with minimal off-targets. For example, biolistics delivery of RNPs may avoid transfer of CRISPR reagent to the next generation and ensure DNA-free editing of plant genomes [182]. Stable integration of plasmids into host genomes increases editing efficiency but produces higher off-targets due to continuous Cas nuclease expression. Off-targets may alter the network of regulatory genes in a host plant, thereby leading to abnormal gene expression. Studies have demonstrated that CRISPR may also cause unacceptable outcomes at target sites, including inadvertent deletions or rearrangements [127,131]. Although CRISPR cassettes can be removed by processes such as selfing and crossing, these studies collectively highlight the need to carefully assess CRISPR-edited crops before releasing them into the environment or the food chain, even if they are transgene-free.

### 5.4. Gene Drives: Forcing Inheritance of a Gene throughout Population

**Figure 9:**
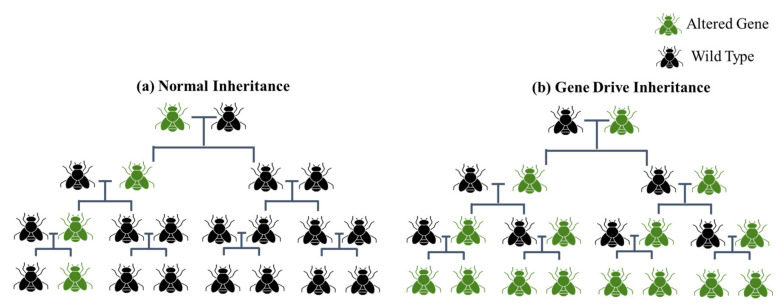
Schematic diagram showing how gene drives linked with CRISPR/Cas systems lead to forced inheritance, spreading the relevant trait throughout the whole population: (**a**) normal Mendelian inheritance; (**b**) gene drive-based inheritance.

CRISPR-based gene drives which use gRNA and Cas9 to copy the drive allele (modified allele linked with gRNA and Cas9) present on one chromosome to the second wildtype homologous chromosome, are always considered transgenic and classified under SDN3, with far reaching impacts on the whole population of a species. CRISPR gene drives are regulated under strict biosafety protocols because they are difficult to recall once released into the environment [127]. Under natural selection, a trait has 50% chance of being inherited throughout a population. A gene drive ensures biased inheritance of a trait throughout a population, bypassing Mendelian inheritance laws (Figure 9). In a CRISPR-mediated gene drive, Cas9 and sgRNA must be present with the drive in a flanking sequence homologous to the DSB site [183]. Gene drives enable transgenic organisms produced under controlled laboratory conditions and released into the environment to spread the desired trait throughout the entire population. In the laboratory, CRISPR gene drives have been found to control vector-borne diseases such as Zika virus disease, dengue fever, and malaria [184]. However, scientists have expressed serious concerns about releasing CRISPR-mediated gene drives into the environment. Any change could become permanent in a species because gene drive traits are difficult to recall once released, although correction drives and suppression drives may help. Any off-target effects resulting from a gene drive would affect the entire population. If a gene drive released for agricultural purposes finds its way into non-target species, this could harm ecosystems, the environment, and potentially also human health. Thus far, no CRISPR-based gene drive has been released into the environment, and no biosafety regulations have been developed to cover them [127,185,186,187]. Gene drives should be very carefully considered for their ecological implications and potential downstream impacts on organisms before release.

### 5.5. Environmental Concerns

Concerns about CRISPR-edited crops are not limited to Cas specificity and off-target impacts on human health. Scientists have also expressed concern about adverse environmental impacts [127]. Although CRISPR/Cas genome-editing systems have been used to improve drought tolerance [188], salt tolerance [189], and nitrogen fixation in plants [190], many see CRISPR crops as having the same potential for negative environmental impacts that GM crops have. For example, the increased use of glyphosate to control weeds in herbicide-resistant GM crops has raised public health and environmental concerns. Although many GM crops offer improved yields and economic benefits, these crops may have serious adverse impacts. GMO-related environmental concerns about pollination, contamination, and reduced biodiversity may also affect CRISPR-edited crops [127].

## 6. CRISPR-Edited Crops and GM Crops: Similarities and Differences

CRISPR is science’s most powerful, adaptable, and precisely targetable tool for site-specific cleavage and precise editing of DNA in living cells [191]. Scientists have used CRISPR to enhance crops for pest resistance [192], disease resistance [193], improved yield [22], and more. In terms of crop improvements, CRISPR can achieve precise, tiny, controlled genome changes that existing genetic engineering tools can never bring about, and the technology is widely considered superior to genetic engineering [170]. CRISPR has the potential to produce improved crops with better social and public acceptance than GM crops have attracted. The most important advantage CRISPR-edited crops have over GM crops is that they do not necessarily contain foreign DNA and are therefore not necessarily considered GMOs [194]. GMOs are produced using methods to tweak the DNA of a living organism and many GM crops include genetic material inserted from foreign organisms, such as *Bacillus thuringiensis* genes in the case of Bt cotton and Bt maize. These modifications have sparked health and environmental controversies, leading to a reluctance in many parts of the world to grow or consume such crops [170]. Creating new GMOs is an expensive and complex process largely restricted to multinational companies. In contrast, CRISPR is simple and cost-effective, thus potentially available to small actors and developing countries [195]. With CRISPR, it is possible to create edited crops that are identical to conventionally-bred crops, and in a much shorter time.

Since the development of genetic engineering in the 1980s, GMOs have been used for many scientific and medical purposes, including the study of basic biological processes and disease mechanisms in animals and plants. Different transformation techniques have been used to create transgenic plants containing genes from non-crossable species. However, the undirected and non-specific approaches used to insert transgenes (genes from non-crossable species) or *cis* genes (genes from crossable organisms) invariably lead to random integration of these genes in the host genome [127]. In contrast, CRISPR methods make small, well-targeted edits at predetermined locations in a plant genome. For example, base editing can replace a single base pair in a genome with great precision without introducing foreign DNA [70], achieving genetic modifications indistinguishable from conventional breeding. Because CRISPR modifications are indistinguishable from natural genetic mutations, many scientists take the view that these CRISPR crops should not be covered by current GMO regulations. Moreover, CRISPR RNPs enable cleaner edits in plants. For example, lipofection has been used to deliver CRISPR RNPs in a non-GMO manner and create transgene-free CRISPR crop plants.

However, although some CRISPR crops are transgene-free and indistinguishable from natural crops, the biological, political, social, and legal differences between CRISPR crops and GMOs are still being debated. A major concern for CRISPR and GM crops is that both use gene markers to aid selection of engineered plants. Although marker genes and Cas9 genes can be removed in subsequent generations of CRISPR-edited crops, concerns persist about whether these genes are completely removed. Identification of desired edits with RNPs and without selection markers is time-consuming and expensive. Critics of CRISPR crops also argue that the processes used to generate CRISPR-edited plants are exactly the same as conventional GM processes. For example, when CRISPR reagents are delivered through Agrobacterium or biolistics, the CRISPR cassette is inserted at a random location in the genome [129]. In the US, which has been deregulating transgene-free CRISPR crops, opponents of GMOs have cautioned about the potential long-term environmental and health implication of CRISPR-edited crops [127,196]. Similar concerns were behind the European Court of Justice (ECJ) ruling that all CRISPR-edited crops in the EU would be regulated under the conventional GMO regulatory framework [197]. Similarities between GM and CRISPR processes are part of the reason why the world community remains divided about the safety and regulation of CRISPR-edited crops.

## 7. Current Regulations for GMOs

### 7.1. Biosafety Regulations for GMOs

Although GM crops provide valuable benefits, many people are concerned about potential risks associated with these plants. For example, gene transfer to non-target plants may pose environmental and health concerns. As well as genes for desirable new traits, antibiotic-resistance genes are sometimes inserted into GM plants to act as markers, enabling rapid selection of plant cells with successful gene transfers. However, there is a small risk these antibiotic-resistance genes could transfer to microorganisms in the human gut. Farmers have expressed serious concern about the development of BT resistance in insects and pests that feed on BT crops [19]. To address concerns about potential risks associated with GM processes and products, many countries have strict regulations governing GMO cultivation and commercialization. Broadly, there are two main approaches to regulatory frameworks for GMOs around the world: (i) product-based regulatory oversight; and (ii) process-based regulations that focus on the techniques used to produce GMOs with new features. The differences in regulatory standards for GMOs might in part be because some countries have not signed the Cartagena Protocol on Biosafety (CPB). The following sections offer a detailed discussion of product– and process-based regulatory frameworks for GMOs.

#### 7.1.1. Product-Based Regulation of GM Plants

Product-based regulatory frameworks focus on new plant varieties without considering the processes used to develop them. These systems take the view that any risk will be specifically associated with the final plant products—not with the techniques used to produce the GM plant. Likewise, any health or environmental concerns are deemed to arise from the final products and not the genetic engineering technologies. Product-based systems are simple, reliable, and compatible with World Trade Organization (WTO) free trade agreements. This avoids the risk of trade restrictions arising from regulations based on specific genetic engineering processes. The US, Argentina, Russia, Australia, and Chile use product-based regulatory systems to control GM crops [127].

#### 7.1.2. Process-Based Regulation of GM Plants

Process-based regulatory frameworks differentiate between GM crops and non-GM crops according to the methods used to generate the particular plant. In contrast to product-based regulations, process-based regulations assume the processes used can influence the potential risks. This assumes that genes are not naturally transferred across species, and any deviation indicates that a plant has been created using a genetic engineering process. The EU uses process-based regulations to control GMOs, including EU direction 2001/18/EC (2001) and EC regulation 258/97 (1997). Process-based assessments are detailed and rigorous, employing a wide range of technically valid tests and observations [122].

#### 7.1.3. Regulations for Plants with Novel Traits

Canada’s regulatory system assesses plants with novel traits (PNTs) developed through conventional breeding or genetic engineering. A PNT is a plant with traits that are either naturally absent in that species or present to a significantly different extent; traits that have not been present in any previously approved product. PNTs are considered to have the potential to significantly affect the Canadian environment [19].

## 8. Ethical Concerns and Public Acceptance of Genome-Edited and GM Crops

The biotechnological sciences are well aligned with the political and economic policies of many countries, and national governments are often major funders of academic biotechnology research. These financial investments are prompted by the expectation of rapid economic growth resulting from the development of new products and services [198].

Serious public and scientific concerns about risks associated with growing GM plants and consuming or using their products have led many countries to regulate the development and commercialization of GM plants. These concerns are largely associated with the insertion of transgenes—genes that would not occur naturally in these plants.

Developers and proponents of CRISPR-edited plants say that these plants and their products do not typically contain any foreign DNA. Their opponents consider crop varieties developed by these technologies to be genetically modified and therefore potentially subject to GMO regulations. As a general rule, new plant breeding technologies (NPBTs) must be regulated to address public concerns and define acceptable biotechnological trajectories in agriculture. However, the potential for CRISPR techniques to bring about heritable changes in a plant genome creates risks that call for globally consistent regulations. A key question is what risks are posed by CRISPR-edited crops if the final products of modified plants are transgene-free. This is also true for some GM products, e.g., canola oil. Public reaction to GM crops is particularly strong in EU countries, where regulations consider all plants modified by NPBTs to be GM even if the resulting plant lines do not contain transgenes [199]. The main causes of public concern are the potential environmental and human health risks. Risk perceptions may also be influenced by social factors, and religious and ethical beliefs such as whether gene scientists are ‘playing God’. People view new crops arising from NPBTs as different from conventional crops and posing unknown environmental risks. Some concerns are due to factors such as limited public understanding of science, lack of trust in developers, inadequate regulations, and poor communication about risks and benefits. Although commercial GM food crops have been grown in countries such as India, Canada, Brazil, US, Argentina, and China since the 1990s, most people in these countries reject food products from GM crops. Along with many EU countries, Japan and New Zealand prohibit cultivation of GM food crops because of public concerns.

If the biotechnology and agricultural sectors and national governments believe genetic engineering can create improved plant varieties that address food security concerns, better public engagement would help bridge the gap between the views of the general public and those of the biotechnology sector. In any case, governments must consider public concerns about the risks and benefits of new crop varieties. New crop varieties with low risks and high benefits are more likely to be publicly acceptable. Education levels vary considerably within and between countries, influencing local knowledge of scientific terms such as cloning, genetic engineering, and biotechnology, and the public’s acceptance of various GM products. This requires further public education, genuine community consultation, transparent regulatory processes for assessing new products, ongoing research into potential environmental and human health risks, and mutually respectful dialogue between legislators, policymakers, and the general public. Any risks associated with new plant varieties from either GM or CRISPR technology could be compared with well-known foods to help people make informed decisions. There are no foods with zero risks. Even plant foods that have been part of human diets for centuries can potentially be toxic. For example, potatoes may contain harmful levels of glycoalkaloids, such as α-chaconine and α-solanine [200].

Another source of consumer concern about GM plants is the risk of transgenes from GM crops transferring to wildtype species, adversely affecting biological diversity and the environment. Although CRISPR/Cas9 genome editing systems can produce transgene-free modifications, the technology is not without risk. Off-target mutations must be carefully investigated, especially in multiplex CRISPR-edited crops in order to enable better risk-benefit assessments.

Additionally, a fear about the potential emergence of new diseases due to modified food products could be answered by continuous risk assessments by the government before bringing the GM plant product to the market. Consumption of GM crops by rodents in some experiments resulted in tumor formation, poor development, and early death in the animals [201,202]. However, in other animal feeding experiments, the use of GM crops has been reported to be safe (EFSA GMO Panel Working Group on Animal Feeding Trials, 2008). But the criticism of the experimental methodologies deployed during these experiments [203,204,205], suggests that research into and monitoring of the potential risks of new products will continue unabated for some time. Overall, there is a significant need to address consumer worries in order to gain widespread public acceptance of genetically-modified crops before commercialization. Governments should create clear and unbiased regulations about GM crops and foster effective communication with developers and the public.

Public acceptance of transgene-free crops may be improved gradually by creating more awareness about CRISPR-based crops, developing trust in safety regulations and developers, and clear comparisons of risks and benefits [206]. Overall, the CRISPR/Cas9 system seems the best strategy to develop improved plant varieties with the least likelihood of concerns. The off-target issues of this technology could be controlled by introducing the nucleases in the form of a rRNP, instead of plasmid DNA [39]. High-fidelity CRISPR variants displaying minimum or even no detectable off-targets at the genome-wide level are also available [207,208]. Additionally, each nuclease or gRNA of the CRISPR/Cas9 system could be evaluated by modern technologies such as whole genome sequencing (WGS) to profile genome-wide off-targets before generating genome-edited organisms (GEOs) [209,210]. On the top of all this, the transgene-free plants produced by CRISPR/Cas9 do not have exogenous DNA in the final product; therefore, the resultant plant species may be able to bypass product-based GMO regulation [206,211]. Transgene-free crops may not cause transgene flow to non-target species and do not need isolated field test and GMO labelling. In some countries, the labelling of GM ingredients, with or without any tolerance level in food product, is mandatory due to ethical values among consumers [206]. On one hand, the labelling of food products produced from GM crops helps to develop public trust; on the other hand, it arguably enhances resistance among specific consumer groups. For example, a field trial in France of the first GM grape vine grafting was disturbed many times by activists, even after legal approval of these trials by a competent government ministry [212]. As a consequence, a request to uncouple safety assessment and environmental risk from the labelling of GEOs has been made by the European Plant Sciences Organization to the European Commission.

DNA tagging into the genome of the crop for the cultivation and marketing of GEOs has been suggested [206]. Nonetheless, DNA tagging requires additional gene modification steps and new GMO regulations, which would be an additional burden on developers and companies and result in higher product costs [213]. Finding an adequate regulatory approach would address safety and legal definitions of genetically-engineered crops. Appropriate regulatory rules would not only increase the public acceptance of GEOs but also require more innovation in agriculture and international trading. Decision-makers need to know the potential economic impact of handling genome-edited products under different regulatory scenarios; this may help them anticipate social perceptions of their decisions [214]. Political decisions should accord with scientific recommendations to avoid ‘over-regulation’ of genome-edited products that might hamper innovation in agriculture and result in adverse impacts on economy and sustainability [215]. We suggest that regulatory policies for GEOs should clearly highlight that the prime objective of genetic engineering in agriculture is to improve food security and consequently contribute to a healthier dietary life, without harming the environment or religious and ethical concepts.

## 9. Global Regulation of GMOs and Genome-Edited Crops

Precise and targeted modifications in the genome loci of plants through advanced molecular biology techniques, such as meganucleases, zinc finger nuclease (ZFNs), TALENs, and CRISPR/Cas9, became popular worldwide due to several potential advantages over traditional plant-breeding techniques for the development of novel plant traits. Even though GM plants could enhance food security significantly for the world’s growing population, their commercial use is restricted to a small number of cultivated crops because of human health and environmental safety concerns.

When the first GM crop was released for field trials in California in 1983, the absence of any regulatory framework for GM plants meant that its potential environmental risks were assessed under the same health protection guidelines as were conventionally-bred plants [216]. As new GM plant traits are adopted at an increasingly rapid pace, there is an urgent need for governments to develop precise and clear regulatory frameworks, which address safety concerns and protect human health and the environment. Many scientists contend that CRISPR edited crops present low risks while at the same time offering major benefits [217]. Where public acceptance is low, this could potentially be addressed by developing consistent regulatory frameworks for assessing risks to human and environmental health. The European Food Safety Authority (EFSA) initially used a ‘comparative safety assessment’ approach to assess GM crops and food risks, drawing on molecular, chemical, and phenotypic data from GM plants and non-GM varieties, and treated genome-edited plants in the same way as GMOs. However, this approach led to over-optimistic conclusions as a result of inadequate data for assessing the adverse biological, toxicological, and ecological effects of GM crops [218]. Anticipated questions and public concerns should be critical points to consider for crop improvement through genome editing. With an almost 100-fold increase in GM crop production in the last 25 years worldwide [219,220], many countries have adopted different regulatory frameworks to fairly address public concerns and control GMOs. Global GMO regulations are categorized as product– or process-based approaches. US, India, Japan, Canada, and Argentina are following a product-based approach in their regulations for GMOs—one where the regulatory framework assesses the characteristics of the end product, irrespective of how it is produced. On the other hand, process-based GMO legislation is based on how the organism is produced. The EU and New Zealand currently regulate GM crops under process-based regulations [206]. These different approaches in the regulation of GM crops may impact global regulation and commercialization of genome-edited (GE) crops. In the following sections, the regulation of GE crops in different countries is discussed and summarized in Table 1.

### 9.1. United States

The US is the world’s largest developer and cultivator of GM crops, accounting for almost 30% of global agricultural biotechnology [19]. The Coordinated Framework for the Regulation of Biotechnology developed in 1984 is still a key regulatory document in the US [236]. GM and CRISPR-edited crops are subject to the same product-based regulation system. New GM plant products are controlled by health, safety, and environmental regulations administered by various agencies, including the Environment Protection Agency (EPA), US Department of Agriculture (USDA), and the Food and Drug Administration (FDA). Each agency has its own responsibilities and assessment criteria. For example, FDA controls medicinal products produced with the help of biotechnology. The EPA assesses pesticides in plants, including GM microbial pesticides such as Bt-toxin. The USDA is responsible for transgenic plants. The USDA’s Animal and Plant Health Inspection Service (APHIS) specifically assesses whether GM plants could pose a pest risk to plants, and assigns either a regulated or non-regulated status. Plants assigned a non-regulated status can be cultivated and transported but not used for food. The APHIS database lists 121 GMOs; these include 19 plant species such as potato, cotton, tomato, corn, and apple that have been deregulated since 1992. GM plants intended as food must also be assessed by the FDA for health and environmental risks. Plant varieties that do not contain foreign DNA from organisms, such as bacteria, viruses, fungi, and insects, are assigned non-regulated status by APHIS under US law [19]. CRISPR-edited plants do not contain foreign or recombinant DNA and are therefore assigned a non-regulated status. For example, the common button mushroom *Agaricus bisporus* was modified by CRISPR/Cas9 to resist browning and spoilage; it obtained a non-regulated status in 2016 [46]. In 2004, the USDA decided that modification of a small number of bases by targeted genome editing was comparable to traditional mutagenesis, so plants modified in this way were unlikely be considered for regulation [237]. However, a memorandum for modernizing the US regulatory system for GE products was passed in 2015. The memorandum states that biotechnology regulations are to be transparent, efficient, and based on the best available science in order to promote public confidence in GE products and biotechnology. To achieve these goals, a new Biotechnology Working Group with members from the Executive Office, FDA, EPA, and USDA was to be established to coordinate with other federal agencies [238]. US authorities view GMOs as ‘novel’ and deserving of patent protection, but also consider them ‘ordinary’ in the sense that safety reviews and testing are not needed. The US assesses the safety of GM products through comparative analysis with their non-GM counterparts. Detailed risk assessments are only triggered if basic plant components—fiber, protein, fats, vitamins, amino acids, ash, minerals, etc.—differ significantly between GM plants and their non-GM counterparts, specifically in the non-GM parent species [218]. Gen-edited plant products already marketed in the US include canola oil from herbicide-tolerant Canola^TM^ plants, high oleic acid soybean oil from Calyno^TM^ soybeans, and starches from waxy maize [19]. The detailed genome editing regulations applicable in the US have been reported by Wolt and Wolf [239].

### 9.2. Canada

Canada is one of the world’s top five cultivators of agricultural biotechnology crops, accounting for an estimated 6.6% of land devoted to such crops around the globe [240]. Like the US, Canada’s GM regulatory framework is product-based, and it does not consider the processes used to create the plants from which the products come. However, all PNTs developed by conventional breeding, traditional mutagenesis, and targeted mutagenesis are assessed under the same risk assessment regulations by the Canadian Food Inspection Agency (CFIA). The CFIA defines novel plant traits as ‘any plant traits that are new to the Canadian environment and could affect environment and human health regardless of whether obtained by conventional, organic, or biotechnological breeding techniques’ [19]. Canada’s assessment of novel plant risks is stringently science-based, focusing on toxicity, off-target impacts, and product allergenicity [241]. PNT regulations apply if the novel trait is expressed at least 20–30% lower or higher than in conventional varieties. Plant varieties can only be registered for commercialization in Canada if the CFIA has granted unconfined environmental release status. Products intended for human food or animal feed purposes must also be assessed by Health Canada or the CFIA Animal Feed Division, respectively [19]. Canada’s regulatory framework has a reputation for delivering timely and consistent judgments [241]. The first GM apple was developed in 2010 using gene silencing but only received approval in 2015. Health Canada and CFIA approved four GM potato varieties in May 2016 after receiving the relevant data in 2015 [241]. In 2016, CFIA approved 100 different biotechnologically-modified plants, according to the Guidance Document Repository (GDR) (http://www.inspection.gc.ca/plants/plants-with-novel-traits/approved-under-review/decision-documents/eng/1303704378026/1303704484236 accessed on 22 October 2021). Product-based legislation appears to foster innovation in agricultural biotechnology [242]. Canada recently approved canola plants generated using oligonucleotide-directed mutagenesis (ODM) to make single-nucleotide mutations in two genes (Canola Event 5715, Cibus Inc., San Diego, CA, USA), suggesting that new plant traits created using targeted genome-editing technologies, such as CRISPR/Cas9, are also likely to be approved. Like other new plant breeding technologies (ZFNs, TALENs, CRISPR), ODM is an alternative approach in genome editing for rapid and precise genetic modifications without inserting transgene in the host genome. Therefore, crops developed through ODM will be evaluated in the same way as CRISPR edited crops.

### 9.3. Latin America

Argentina defines GMOs as organisms ‘having a novel combination of genetic material obtained through modern biotechnological techniques’ [214]. However, if a final product has no transgenes, regardless of whether the plant was created using transgene techniques, it will be classified as non-GM. Argentina’s regulatory system could be classified as both product-based and process-based, depending on the specific event. Because Argentina’s regulatory framework for GM crops uses case-by-case assessment, each new plant modification can be individually regulated if necessary [243]. This approach for gene-editing was developed following intensive discussions between regulators and policymakers in 2012. It is consistent with the CPB, even though Argentina has not adopted the Protocol. In May 2015, Argentina was the first country to make public its regulatory resolutions on GM crops created by NPBTs.

In 2017, with the aim of recognizing GM crops and working towards consistent approvals in Latin America, agriculture ministers from Argentina, Brazil, Chile, Paraguay, and Uruguay signed a declaration [19] on NPBTs [244]. Eight out of 12 Latin American countries agreed to a case-by-case assessment policy that excluded some gene-edited products from strict GM regulation and embraced biotechnology in the region [245].

Other Latin American countries, including Ecuador, Venezuela, and Peru, do not allow commercialization of GM crops. Ecuador declared itself a transgenic-free territory in 2008, but later allowed the entry and cultivation of GM seeds for research purposes only [246]. In May 2019, Ecuador implemented Executive Decree No. 752 to accommodate NPBTs, excluding organisms that do not contain foreign or recombinant DNA from risk assessment [245]. Peru (2011) and Venezuela (2015) have prohibited the entry and cultivation of GM crops and seeds, respectively enacting a 10-year legislative moratorium [247] and the Seed Law [19] on GM crops. Peru’s 10-year legislative moratorium on GM crops was extended for a further 15 years by the Peruvian Congress before the expiration of the moratorium in 2021 [19]. The moratorium gives Peru time to enact regulations governing adoption of NPBTs, but as yet there are no defined regulations in place for CRISPR-edited crops [248]. Venezuela imports GM soybean and maize crops from the US, Argentina, and Brazil [19]. However, Venezuela considers GMO release to be a major cause of biodiversity loss and has prohibited the release of GM crops into the environment [221]; GM plants and seeds are banned, even for research purposes [19].

Like Argentina, Chile uses a case-by-case approach for NPBT-derived plants. Chile’s Agricultural and Livestock Service (SAG) assesses GM plant varieties and products according to whether foreign or recombinant DNA is present. So far eight genome-edited products have been assessed as non-GMO [224]. Chile is the world’s ninth-largest exporter of GM seeds [224], with SAG controlling GM seed production, imports, exports, and field trials. However, GM seeds cannot be cultivated as domestic product in Chile. Conversely, Chile does not restrict GM food and feed imports from other countries, including GM maize and soybeans from Brazil [225].

### 9.4. European Union

The EU uses process-based GMO legislation. EU member states assess environmental and food safety risks on the basis of the processes used to create a particular GMO (plant, animal, or microorganism), and do not consider the final products of that GMO. In 2018, the European Court of Justice (ECJ) defined a GMO as an organism with genetic material altered by any non-natural means (directed or random mutagenesis), including NPBTs such as CRISPR/Cas-directed mutagenesis, irrespective of size and type of any DNA alteration [19,226]. According to ECJ case C-520/1650, all organisms created using genome-altering techniques SDN1, SDN2, and SDN3 must be regulated under the regulatory framework for GMOs [180]. The few exemptions include mutation breeding-based techniques already in use before the Directive (2001/18/EC) entered into force in 2001 [227]. Since the first EC directives on GMO usage and deliberate environmental release were issued in 1990—90/219/EC [228] and 90/220/EC [229]—these have been revised many times. The current directives are 2001/18/EC and 2009/41/EC [230]. All 27 EU member states regulate GM food and feed products according to Regulation (EC) No 1829/2003, which aims to achieve a high level of protection for human, animal, and environmental health. Although NPBTs have numerous applications in agriculture, they are the subject of rigorous ongoing debate as regards scientific and ethical issues. Genome-editing techniques, especially CRISPR/Cas9, became highly controversial in 2015 when they were applied to human embryos by scientists in China [231]. By early 2015, several European-based environmental non-government organizations (eNGOs) had signed a letter calling on the European Commission to ban all new breeding techniques (NBTs) within the EU. Many EU member states banned GM crop cultivation following the introduction of a safeguard clause in 2015 [222]. Although many countries in the Americas have incorporated these new technologies, the EU has rejected the technology entirely, thereby losing billions of agricultural research and development funding [241]. In the last 25 years, two plant modifications have been approved, but only one is routinely cultivated, an insect-resistant maize (MON810) grown in Spain and Portugal [19]. Having originated in the 1990s, the EU regulatory framework does not accommodate more recent plant-breeding techniques. Early this year, in 2021, the Council of the European Union requested a proposal and study on the status of new genomic techniques to be submitted by April 2021 [232]. The aim was to support the evolution of regulatory laws in the region. Currently, EU member states can import approved GM food and feed products but cannot cultivate GM crops. The legal status of GMOs and their products derived from NPBT technologies is still unclear in the EU.

Like EU countries, Norway and Switzerland have national legislation which restricts GM crop cultivation. However, Switzerland imports GM crop products for animal feed purposes [19]. In 2016, when a temporary Swiss moratorium on GM crop cultivation and processing was extended for the third time, the Swiss Cabinet recommended the creation of a separate zone for Swiss GM crops from 2021. In contrast, although Norway neither cultivates nor imports GM food or feed crops, GM crops are legally allowed in the country under the Norwegian Gene Technology Act. Thus far, the only GM product or environmental release approved by Norway’s Food Safety Authority is a single species of ornamental purple carnation [19]. In addition to following EU health and environmental safety criteria for GMOs and their products, Norwegian regulations require three non-safety assessments: for social benefits, sustainability, and ethical soundness. In recent years, the Norwegian Biotechnology Advisory Board proposed relaxing some of Norway’s strict regulations governing GM plant products and the environmental release of GM plants [233], indicating an effort to reduce the gap between GMO science and law.

### 9.5. India

India is the world’s largest Bt cotton producer [233]. Its Ministry of Environment, Forest and Climate Change (MoEFCC) implemented the country’s Environmental Protection Act (EPA) in 1986. India’s top regulatory body for GM crops is the Genetic Engineering Appraisal Committee (GEAC), which was set up under EPA and MoEFCC. All GM crops need GEAC approval before they can be commercialized for public consumption. MoEFCC also follows the CPB, and has established institutes to evaluate biosafety regulations for GM crops; one such institute is the Biosafety Clearing House set up in 2017 [234,235]. The CPB sets out guidelines necessary for environmental protection and safe human consumption of all living GMOs, including GM crops. India’s regulations were initiated in 1982, after the foundation of a National Biotechnology Board [223]. MoEFCC introduced the regulations under EPA in 1986, which was altered to become ‘the rules for the manufacture, use, import, export and storage of hazardous microorganisms, genetically engineered organisms’ in 1989 [234]. The EPA referred to hazardous microorganisms and genetically engineered organisms as ‘hazardous substances’. Committees established under India’s EPA include the Recombinant DNA Advisory Committee (RDAC), Review Committee on Genetic Manipulation (RCGM), GEAC, and various institutional biosafety committees (IBSC) [234]. The Parliament of India has passed three acts governing the development, approval, and commercial release of GM crops. These include the EPA 1986, administered by the Ministry of Environment, Forest and Climate Change (MoEFCC), the Seeds Act 1966 & Seeds Order (an historical seeds order replaced by plant quarantine order under the Ministry of Agriculture and Food Safety) [249] and the Food Safety and Standards Act 2006 under the Ministry of Health and Family Welfare [250].

India also established the Biotechnology Regulatory Authority of India (BRAI) for assessing modern GMOs and promoting farmer awareness of modern GM products. To date, India’s only GM crop approved for commercial farming is Bt cotton, which has been approved for cultivation of seeds, fiber, and feed production. Bt eggplant was approved in 2009, but suspended by MoEFCC in 2010. Improvement of GM crops for the public sector—mostly by government agencies, including CRIDA, ICAR-IIOR (Hyderabad), ICAR-CICR (Nagpur), CPRI (Shimla), and UPCSCR (Shahjahanpur)—is typically undertaken for pest resistance, tolerance to herbicides and drought, increased nutritional value, and higher yields. Public-sector GM crops include banana, cauliflower, chickpea, cabbage, mustard, wheat, papaya, potato, cassava, tomato, rice, and watermelon. Private companies such as Mossanto, JK Agri Genetics, Nath Seeds, and Bayer Bioscience focus more on cabbage, corn, mustard, okra, peas, tomatoes, cotton, and cauliflowers. As a result of disputes in relation to state government approvals, field trials have only been conducted for cotton, corn, and rice [234,249].

### 9.6. China

China has successfully grown Bt cotton since 1997 and was the world’s sixth-largest cultivator of GM crops in 2013 with four million hectares under cultivation.

In China, the Ministry of Agriculture (MoA) provides general regulations for GM crops, mainly addressing the safety of primary GM crop production. Until the early 2000s, China had no specific regulations for safety assessment or testing of GMOs for human consumption. In 2001, the Ministry of Health (MoH) began to fill the gap. The 2002 Rules on Hygiene Administration of Genetically Modified Food required GM plants, animals, and microorganisms to be assessed for safety and nutritional value. In 2007, these rules were replaced by the Rules on Administration of Novel Food Materials. The Food Safety Law 2015 emphasized the importance of proper labeling of GM foods during production and sale. If these rules are violated, customers can claim punitive damages 10 times the price of original product [251]. The Ministry of Environmental Protection (MEP), founded in 2018, bears primary responsibility for implementing and developing Chinese regulations and biosafety protocols for GM crops [19].

In 2011, the Ministry of Agriculture implemented rules that are used to protect new GM plant varieties, including oilseed, hemp, sugar crop, tobacco, mulberry, grains, cotton, ornamental plants, herbal medicinal plants, rubber tree, edible algae, and fungi [252]. To date, China’s Ministry of Agriculture and Rural Affairs has approved more than 64 submissions for cultivation of GM crops, including maize, soybeans, cotton, tomato, rice, papaya, *Petunia alba*, sugar beet, Argentine canola (*Brassica napus*), and sweet pepper [253]. In 2017, around 2.8 million hectares of land was devoted to growing Bt cotton and GM papaya. Although China has approved other GM crops, such as Bt rice and ring spot-resistant GM papaya, they are not cultivated commercially. China permits GM maize and soybean imports for animal feed.

### 9.7. Japan

Japan is among the world’s largest importers of GM crops, such as corn, sugar beet, maize, and soybean [254]. In 2018, Japan was second only to the US for total number of GM approvals [19]. A further 141 GM crop varieties were approved in 2020 [255]. Japan has an unusual approach to the regulation of GM crops. Regulatory laws governing food safety and quality for GM crops in Japan include the Food Sanitation Act 1947 and the Act on Standardization and Proper Quality Labeling of Agricultural and Forestry Products 1950, which were consolidated in 2006. The Food Sanitation Act was revised several times before it came into effect in 2001. After adopting the CPB in 2003, Japan brought in the Act on the Conservation and Sustainable Use of Biological Diversity through Regulations on the Use of Living Modified Organisms (LMOs), also known as the Japanese Cartagena Act [256,257]. Japan has several GMO approval authorities. GMOs for human consumption are approved by the Ministry of Health, Labor and Welfare (MHLW). The Ministry of the Environment has final authorization for GMO usage for livestock feed. Commercial crops are approved by the Ministry of Agriculture, Forestry and Fisheries (MAFF). To date, Japan has not permitted any GM cultivation apart from blue roses [258], but it allows GM ingredients and crops to be imported for food, feed, and processing purposes.

All GM foods must be assessed for safety by the Food Safety Commission of Japan (FSCJ) before they can be distributed in commercial or domestic markets. Under Japanese legislation, official bodies assess potential environmental risks associated with GM grains, including accidental mixing with commercial non-GM grains [259]. During consideration of potential new GM crops, FSCJ informs the general public and allows them to offer feedback to FCS. When assessments are complete, the final decision is made by MHLW in consultation with FSCJ.

Approved GM crops and products must meet mandatory labeling protocols. Labeling legislation was initiated in 2001 under the Act on Standardization and Proper Quality Labeling of Agricultural and Forestry Products. GM products are divided into three categories for labeling: GM, GMOs not segregated, and non-GM. Since traceability is not mandatory, only products with raw ingredients that contain GM DNA are labeled as GM. Products do not need to be labeled as GM if the GM DNA is not preserved in the end product. Such products are usually meant for animal feed [254].

### 9.8. Australia and New Zealand

Australia and New Zealand began introducing strict GMO precautions and regulations in 2001. Food Standards Australia New Zealand (FSANZ) regulates marketing of GM food products in both countries. As in the EU, FSANZ has one of the world’s most stringent food safety regulations. FSANZ stipulates mandatory approval and labeling for GM crop production and GM foods for domestic markets. In Australia, however, GM crop cultivation is regulated by a separate agency, the Office of the Gene Technology Regulator, and the country has grown Bt cotton since 1996 and GM canola varieties since 2004 [260]. New Zealand initially imposed a moratorium on GM production, but lifted this in 2003 when the New Organisms and Other Matters Act came into being [261]. In 2019, Australia clarified its regulations for genome-edited crops by amending its Gene Technology Regulations 2001. Under this amendment, SDN1 plants would not automatically be considered GMOs. SDN1 crops would no longer come under the Gene Technology Act 2000 and would instead be regulated by the Department of Agriculture, Water and Environment. In 2014, New Zealand briefly considered but then rejected excluding gene-edited organisms from GMO legislation [262], and has continued to regulate gene-edited organisms under the Hazardous Substances and New Organisms Act (HSNO), administered by New Zealand’s Environmental Protection Authority.

In the authors’ collective opinion, countries that adapt up-to-date GMO regulatory frameworks and biosafety laws to accommodate gene-edited crops and their products stand to reap gains in domestic agriculture and commercial success. Global harmonization of different GMO regulations and standards could bring the development of biosafety laws for gene-edited crops a step closer. Such regulatory harmonization could be achieved through sharing experiences and technical expertise, considering political and societal interests, and identifying shared opinions. Most countries assess SDN1 plant products under regulations that apply to conventionally-bred plant varieties rather than GMO regulations [157]. The assessment of SDN2 plant varieties under GMO regulations [263] could affect global harmonization regulatory efforts.

## 10. Prospects

CRISPR-derived biotechnologies have been adopted universally by academic and industrial research groups for improving plant genomes. Agricultural and food scientists have used CRISPR/Cas technology to improve plant defenses against viral and insect attacks, environmental stresses, and bacterial and fungal diseases. CRISPR has enabled precise, site-specific changes in plant genomes without inserting transgenes into host genome—changes not achievable using GM technology. CRISPR has quickly become one of the most widely-used techniques for rapid development of new crop varieties with superior traits. CRISPR technology is viewed as a game changer in varietal development and plant genetic improvements due to its precision, simplicity, versatility, efficiency, and multiple genome-editing abilities. Multinational companies in the US and elsewhere are racing to develop CRISPR-edited crops with desirable traits and bring them onto international markets. However, despite worldwide acknowledgment of CRISPR’s tremendous potential for plant genetic improvements, the technology’s future will be determined by how CRISPR-edited crops and their products are to be regulated.

The commercialization of CRISPR-edited plant crops faces many hurdles, including regulation, public acceptance, and whether such crops are classified as GMOs or non-GMOs. Popular protocols that employ Agrobacterium and biolistics transformations and use plasmids as CRISPR reagents lead to permanent integration of Cas9 and sgRNA into host genomes and may thereby cause off-target impacts. In addition, although CRISPR is generally considered a highly specific technique, any non-specific binding of sgRNA in the genome may also lead to off-target mutations. Using RNPs as CRISPR reagents to generate transgene-free plants is appealing, but editing efficiency is not impressive. RNP applications are also limited by transformation and regeneration difficulties in some crop species. The current global regulatory landscape for CRISPR-edited plants is patchy. As with GMOs, different countries have adopted different regulations for assessing CRISPR-edited plants. The US, Argentina, Columbia, Chile, and Brazil are facilitating development and deregulation of CRISPR-edited plants if they are indistinguishable from natural mutations. EU and New Zealand consider CRISPR-edited plants in the same light as GMOs, and strictly regulate their commercialization. Many of the world’s less-developed countries have not yet devised regulatory systems for assessing CRISPR-edited plants.

In our opinion, it is essential to distinguish CRISPR technologies from earlier GM methods. The development, commercialization, and changing public perceptions of GMOs and CRISPR-edited plants present an opportunity to raise the level of international dialogue about regulating CRISPR-derived crops and address major global issues such as food security, sustainable development, and climate change. The development of a universal and scalable regulatory system for CRISPR crops will require open and unbiased dialogue between scientists, governments, commercial interests, consumers, and all forms of media. With multiplex genome-edited crops and gene-derived products likely to soon reach the market, regulations are essential to avoid adverse environmental impacts. The world needs all stakeholders to constructively engage in discussing the past, present, and future of CRISPR-edited plants

## Figures and Tables

**Table 1:** Details of worldwide commercially approved genome-edited crops and legislation on the release of gene-edited plants.

Continent	Country	Regulatory Agencies	GMO Commercial Cultivation Area (Million Hectares)	Approved Genome Edited Crops	Approve Year	Regulation Governing the Release of Gene-Edited Crops	SDN1	SDN2	SDN3	References
North America	US	USDA, APHIS, FDA, and EPA	75	Corn	2018	Coordinated Framework for Regulation of Biotechnology, New SECURE rules (2020)	Deregulated	Deregulated	Case by case	[221,222,223]
				Tomato	2018					
				Soybean	2017					
				Mushroom	2016					
				Flax	2017					
				Non browning apple						
	Canada	Canadian Food Inspection Agency (CFIA)	11	Non browning Potato	2016	Directive 94–08 (Dir 94–08) Assessment Criteria for Determining Environmental Safety of Plants with Novel Traits	Novelty based regulation	Novelty based regulation	Novelty based regulation	[222,223,224]
				Herbicide resistant canola	2015					
Latin America	Argentina	Argentine Biosafety Commission (CONABIA)	24.5	HB4 drought resistant wheat	2020	Resolution No. 173/15 (2015)	Deregulated	Deregulated	De-regulated (If not transgenic)	[222,223,225]
	Brazil	National Technical Commission for Biosafety (CTNBio)	53	No approved crops		Normative Resolution No. 16 (2018)	Deregulated	Deregulated	De-regulated (If not transgenic)	[222,223,226]
	Chile	Ministry of Agricultural and Livestock Services (SAG)	Less than 1	No approved crops		Introduction of methodological procedure (2017)	Deregulated	Deregulated	De-regulated (If not transgenic)	[195,223,227]
	Columbia	Colombian Agricutural Institute (ICA)	0.1	No approved crops		Resolution No. 00029299 (2019)	Case by case	Case by case	De-regulated (If not transgenic)	[195,223,228]
	Honduras	National Committee of Biotechnology and Biosecurity (NCBB)	Less than 1	No approved crops		Agreement SENASA 008-2019 (2019)	Case by case	Case by case	De-regulated (If not transgenic)	[195,223,229]
Asia and the Pacific	Australia	Food Standards Australia New Zealand (FSANZ)	0.9	No approved crops		Gene Technology Act (Measures No. 1) to regulations (2019)	Deregulated	Deregulated	Regulated	[195,223,230]
	China	National Biosafety Committee (NBC), Ministry of Agriculture and Rural Affairs (MARA)	2.8	No approved crops		Administrative rules for safety of agricutural GMOs	Under development	Under development	Under development	[195,223,231]
	India	Indian Ministry of Science and Technology (2020), Genetic Engineering Appraisal Committee (GEAC)	11.4	No approved crops		Regulatory Framework and Guidelines for Risk Assessment (2020)	Under development	Under development	Under development	[195,222,223]
	Japan	The Ministry of Agriculture, Forestary and Fishries (MAFF)	No	Tomato	2021	GMO as defined under Cartagena Act (2019)	Deregulated	Deregulated	Regulated	[223,232]
	New Zealand	Food Standards Australia New Zealand (FSANZ)	No	No approved crops		Hazardous Substances and New Organisms Act (1998) after court decision NZHC 1067 (2014)	Regulated	Regulated	Regulated	[223,233]
	Pakistan	National biosafety committee	2.9	No approved crops		Pakistan Biosafety Rules, 2005	Under development	Under development	Under development	[167,234]
European Union	Only Spain and Portugal		0.1	No approved crops		Directive 18/2001/EC (2001) after court decision in case C-528/16	Regulated	Regulated	Regulated	[24,195,222,235]
